# Immediate Antihypertensive Treatment for Patients With Acute Ischemic Stroke With or Without History of Hypertension: A Secondary Analysis of the CATIS Randomized Clinical Trial

**DOI:** 10.1001/jamanetworkopen.2019.8103

**Published:** 2019-07-31

**Authors:** Rui Zhang, Chongke Zhong, Yonghong Zhang, Xuewei Xie, Zhengbao Zhu, Aili Wang, Chung-Shiuan Chen, Yanbo Peng, Hao Peng, Qunwei Li, Zhong Ju, Deqin Geng, Jing Chen, Liping Liu, Yilong Wang, Tan Xu, Jiang He

**Affiliations:** 1 Department of Epidemiology, School of Public Health and Jiangsu Key Laboratory of Preventive and Translational Medicine for Geriatric Diseases, Medical College of Soochow University, Suzhou, China; 2 Department of Epidemiology, Tulane University School of Public Health and Tropical Medicine, New Orleans, Louisiana; 3 Department of Neurology, Beijing Tiantan Hospital, Capital Medical University, Beijing, China; 4 Department of Neurology, Affiliated Hospital of North China University of Science and Technology, Hebei, China; 5 Department of Epidemiology, School of Public Health, Taishan Medical College, Shandong, China; 6 Department of Neurology, Kerqin District First People’s Hospital of Tongliao City, Inner Mongolia, China; 7 Department of Neurology, Affiliated Hospital of Xuzhou Medical College, Jiangsu, China; 8 Department of Medicine, Tulane University School of Medicine, New Orleans, Louisiana

## Abstract

**Importance:**

Clinical trials have generally shown a neutral effect of early blood pressure (BP) decreases on clinical outcomes after acute ischemic stroke. Whether the effect of early antihypertensive therapy differs for patients with ischemic stroke with or without prior hypertension is unclear.

**Objective:**

To investigate the association between immediate antihypertensive treatment and patient outcomes according to the presence or absence of hypertension before stroke onset.

**Design, Setting, and Participants:**

This study was a prespecified subgroup analysis of the China Antihypertensive Trial in Acute Ischemic Stroke (CATIS), a multicenter, single-blind, blinded end-points randomized clinical trial of 4071 patients with acute ischemic stroke and elevated systolic BP. Patients were recruited from August 2009 to May 2013, and this statistical analysis was performed using the intention-to-treat population from January to October 2018.

**Interventions:**

Participants were randomly assigned to receive antihypertensive treatment (aimed at decreasing systolic BP by 10%-25% within the first 24 hours after randomization, achieving systolic BP <140 mm Hg and diastolic BP <90 mm Hg within 7 days, and maintaining this level during hospitalization) or to the control arm (discontinued all antihypertensive medications).

**Main Outcomes and Measures:**

Primary outcome was a combination of death and major disability (modified Rankin Scale score ≥3; range 0-6, with higher values indicating greater disability) at 14 days or hospital discharge.

**Results:**

In total, 2038 patients were randomized to receive antihypertensive treatment, and 2033 patients were randomized to the control group. The mean (SD) age was 62.0 (10.9) years, and 2604 participants (64.0%) were men. At day 14 or hospital discharge, the primary outcome was not different between the treatment and control groups among patients with or without prior hypertension (*P* = .97 for homogeneity): odds ratios (ORs) associated with treatment were 1.00 (95% CI, 0.87-1.16) for patients with prior hypertension and 1.00 (95% CI, 0.75-1.32) for patients without. Early antihypertensive treatment was associated with different rates of 3-month recurrent stroke (patients with hypertension: OR, 0.44; 95% CI, 0.25-0.77 vs without hypertension: OR, 3.43; 95% CI, 0.94-12.55; *P* = .005 for homogeneity) and vascular events (patients with hypertension: OR, 0.66; 95% CI, 0.43-1.02 vs those without hypertension: OR, 1.91; 95% CI, 0.75-4.83; *P* = .04 for homogeneity) by hypertension history.

**Conclusions and Relevance:**

Among patients with acute ischemic stroke, early antihypertensive treatment was not associated with different death and major disability outcomes by hypertension history. However, early antihypertension therapy was associated with a decreased rate of recurrent stroke among patients with a history of hypertension and may inform future studies in the optimal approach to hypertension management in the setting of acute ischemic stroke.

**Trial Registration:**

ClinicalTrials.gov identifier: NCT01840072

## Key Points

**Question:**

Is early antihypertensive therapy associated with different outcomes among patients with ischemic stroke with or without a history of hypertension?

**Findings:**

In this prespecified subgroup analysis of the China Antihypertensive Trial in Acute Ischemic Stroke randomized clinical trial, early antihypertensive treatment was not associated with different death and major disability outcomes among 4071 patients with ischemic stroke with or without hypertension. However, early antihypertensive therapy was associated with significantly decreased 3-month rates of recurrent stroke among patients with prior hypertension.

**Meaning:**

These findings suggest that continuing antihypertensive treatment may decrease the risk of recurrent stroke among patients with acute ischemic stroke and prior hypertension, and the decision to decrease blood pressure should be based on individual clinical judgment, requiring caution for those without prior hypertension.

## Teaser

This prespecified subgroup analysis of the China Antihypertensive Trial in Acute Ischemic Stroke (CATIS) randomized clinical trial evaluates the association between immediate antihypertensive treatment and patient outcomes among patients with acute ischemic stroke with vs without a history of hypertension.

## Introduction

Stroke is a major cause of death and the leading cause of serious, long-term neurologic disability in adults worldwide.^1^ Blood pressure (BP) is recognized as an important determinant of the risk of initial stroke among individuals with hypertension as well as among those without hypertension.^2,3^ However, different effects of antihypertensive intervention may occur in individuals with or without hypertension.^4,5,6^ Clinical trial findings support that decreasing BP decreases the risk of cardiovascular disease in hypertensive patients, but inconsistent effects have been observed among persons with prehypertension or normal BP.^4,5,6,7^

Patients with acute ischemic stroke often also have an acute hypertensive response, which affects up to 80% of patients.^8,9^ The initial hypertensive response is self-limiting; it is most marked in the first few hours following the onset of cerebral ischemia and resolves during the next several days.^10,11,12^ Although the benefit of decreasing BP for primary and secondary prevention of stroke has been established, the effect of immediate antihypertensive treatment of patients with acute ischemic stroke and elevated BP has been uncertain.^12,13,14^ In addition, whether ongoing antihypertensive therapy should be continued or stopped among patients with acute ischemic stroke and prior hypertension still needs to be resolved. On one hand, BP-decreasing treatment may reduce vascular damage, cerebral edema, and hemorrhagic transformation of the cerebral infarction. It may then help to prevent secondary injury and to hasten the transition to long-term antihypertensive therapy. On the other hand, early BP reduction may also decrease cerebral perfusion of the ischemic tissue and further increase the size of the cerebral infarction.^10^ This has been the main concern about decreasing BP early after stroke, especially for patients who are hypoperfused or nonhypertensive and have ischemic stroke. Therefore, whether it is necessary to have different BP management strategies for patients with acute ischemic stroke with or without a history of hypertension before stroke onset is unclear. Previous clinical trials have indicated that history of hypertension does not significantly modify the effect of early decreasing of BP after stroke.^15,16,17^ However, these trials have been conducted in patients with acute intracerebral hemorrhage or either acute ischemic or hemorrhagic stroke. To our knowledge, there are no published clinical trials with sufficient statistical power to address the modification of hypertension history on the effect of early BP decreasing for clinical outcomes among patients with acute ischemic stroke.

The China Antihypertensive Trial in Acute Ischemic Stroke (CATIS) was a multicenter randomized controlled trial designed to test the effect of decreasing BP within the first 48 hours after the onset of an acute ischemic stroke on death and major disability.^14^ Here, we report on a prespecified subgroup analysis of CATIS on the effect of antihypertensive treatment during the acute phase of ischemic stroke on death and disability among patients with or without prior hypertension.

## Methods

### Trial Design and Participants

The multicenter, single-blinded, blinded end-points randomized clinical trial CATIS was conducted in 26 hospitals across China from August 2009 to May 2013. Details on the rationale, design, and main results are published elsewhere.^14^ In brief, we recruited 4071 patients aged 22 years or older who had ischemic stroke confirmed by computed tomography or magnetic resonance imaging of the brain within 48 hours of symptom onset and who had systolic BP between 140 and 220 mm Hg. Patients with systolic BP of 220 mm Hg or higher, diastolic BP equal to or higher than 120 mm Hg, severe heart failure, acute myocardial infarction or unstable angina, atrial fibrillation, aortic dissection, cerebrovascular stenosis, resistant hypertension, or deep coma were excluded. In addition, patients treated with intravenous thrombolytic therapy at baseline were excluded because of different requirements for BP reduction (see trial protocol in Supplement 1). This study followed the Consolidated Standards of Reporting Trials (CONSORT) guideline for randomized clinical trials.

This study was approved by the institutional review boards at Tulane University, New Orleans, Louisiana, in the United States and at Soochow University in Suzhou, Jiangsu Province, China, as well as by the ethical committees at the 26 participating hospitals. All participants provided written informed consent; they did not receive financial compensation.

### Intervention

In the CATIS trial, participants were randomly assigned to receive antihypertensive treatment or to the control group. Randomization was conducted centrally and was stratified by participating hospitals and use of antihypertensive medications. The randomization schedules were generated using PROC PLAN in SAS and concealed until an eligible participant was ready for enrollment. The treatment group received antihypertensive treatment aimed at decreasing systolic BP by 10% to 25% within the first 24 hours after randomization, decreasing systolic BP to 140 mm Hg or less and diastolic BP to 90 mm Hg within 7 days, and maintaining this level of BP control during the remainder of a patient’s hospitalization. Multiple antihypertensive agents were used individually or in combination in the intervention group to achieve the targeted BP decrease according to a prespecified treatment algorithm (eFigure in Supplement 2). Patients in the control group discontinued previously prescribed antihypertensive medications while they were hospitalized. After their hospital discharge, patients in both groups were prescribed antihypertensive medications according to clinical guidelines. The treatment was designed to test for BP treatment strategy rather than the efficacy of specific antihypertensive drugs.

### Measurements

Demographic characteristics and medical histories were collected at the time of enrollment. Stroke severity was assessed at baseline using the National Institutes of Health Stroke Scale by trained neurologists.^18^ History of hypertension was defined as a *yes* answer to the question “have you been told by the doctor that you have high BP?” or to the question “are you currently taking a drug to lower BP?” Baseline BP measurements were obtained by trained nurses according to a common protocol adapted from procedures recommended by the American Heart Association. The BP was measured with the participant in a supine position using a standard mercury sphygmomanometer and 1 of 4 cuff sizes based on participant arm circumference. After randomization, 3 BP measurements were obtained every 2 hours for the first 24 hours, every 4 hours during the second and third days, and 3 times a day thereafter until hospital discharge or death.

### Outcome Assessment

The primary outcome was a combination of death and major disability (modified Rankin Scale [mRS] score ≥3; range 0-6, with higher values indicating greater disability) at 14 days or hospital discharge. The secondary outcome was a combination of all-cause mortality and major disability at the 3-month posttreatment follow-up visit. Major disability was defined as a score of 3 to 5 on the mRS at 14 days and at 3 months after randomization.^19^

Other secondary outcomes included an ordered 7-level categorical score of the mRS for neurologic functional status, vascular events (ie, vascular deaths, nonfatal stroke, nonfatal myocardial infarction, hospitalized and treated angina, hospitalized and treated congestive heart failure, and hospitalized and treated peripheral arterial disease), recurrent fatal and nonfatal stroke, and all-cause mortality assessed at the 3-month posttreatment follow-up visit. Participants were followed up in person at month 3 by trained neurologists and research nurses unaware of treatment assignment. Death certificates were obtained for deceased participants, and hospital data were abstracted for all vascular events. A trialwide outcomes assessment committee, blinded to treatment assignment, reviewed and adjudicated vascular events based on criteria established in the Antihypertensive and Lipid-Lowering Treatment to Prevent Heart Attack Trial (ALLHAT).

### Statistical Analysis

Data were analyzed using an intention-to-treat analysis. Baseline characteristics were compared between the antihypertensive treatment and control groups in patients with or without prior hypertension. The difference in means between the antihypertensive treatment and control groups was tested using *t* tests, percentages were assessed using χ^2^ tests, and medians were assessed using the Wilcoxon rank sum test. The proportions of participants with primary and secondary outcomes at 14 days or at discharge and at the 3-month posttreatment follow-up were compared between the antihypertensive treatment and control groups using a χ^2^ test at a 2-sided α level of 5%, without correction for multiple comparisons. Logistic regression analysis was used to estimate unadjusted odds ratios (ORs) and 95% CIs associated with antihypertensive treatment compared with no antihypertensive treatment. In addition, ordinal logistic regression was used to estimate the effect of BP decrease on the full range of the mRS.^20^ Heterogeneity of the treatment effect on primary and secondary outcomes according to the presence of hypertension was assessed by adding an interaction term in the logistic regression models. In a sensitivity analysis, we further adjusted age, sex, baseline systolic BP, body mass index (calculated as weight in kilograms divided by height in meters squared), history of diabetes, cigarette smoking, stroke subtype, baseline National Institutes of Health Stroke Scale score, and time from onset to randomization to test the robustness of our findings. Data analyses were performed from January to October 2018, using SAS, version 9.4 (SAS Institute Inc).

## Results

### Study Participants

**Figure 1.**
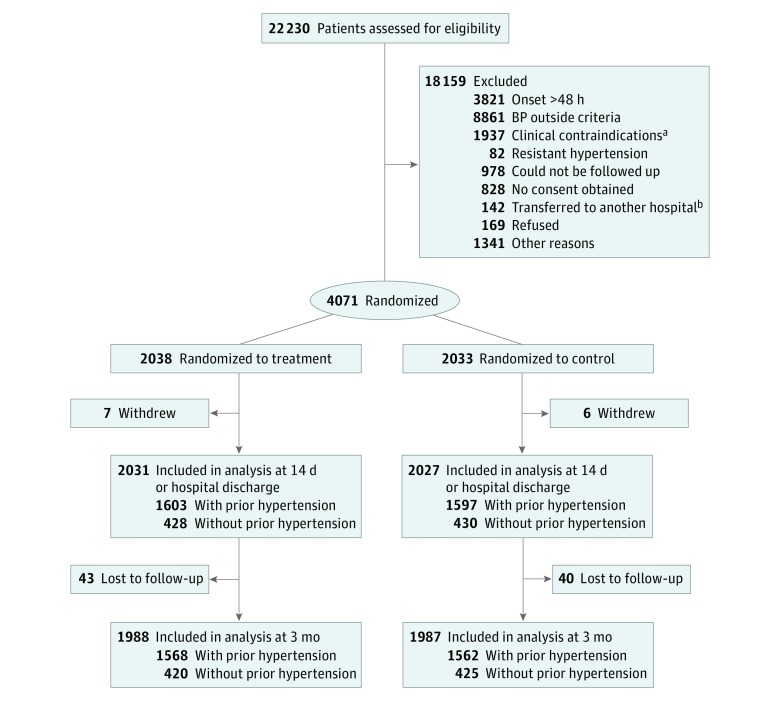
Study Participant Flowchart BP indicates blood pressure. ^a^Individuals with severe heart failure (New York Heart Association class III and IV), myocardial infarction, unstable angina, atrial fibrillation, aortic dissection, cerebrovascular stenosis, or in a deep coma. ^b^Eligible at screening visit but transferred to another hospital before randomization.

A total of 22 230 patients with acute ischemic stroke were screened from August 2009 to May 2013, and 4071 participants were recruited in the CATIS trial. Of these, 2038 were randomized to receive antihypertensive treatment, and 2033 were randomized to the control group (Figure 1). The mean (SD) age was 62.0 (10.9) years, 2604 participants (64.0%) were men, and 3170 patients (77.9%) had thrombotic strokes. There were 3209 participants (78.8%) who had a history of hypertension (1610 were randomly assigned to receive antihypertensive treatment and 1599 were assigned to the control group), and 862 with no history of hypertension (428 were randomly assigned to receive antihypertensive treatment and 434 were assigned to the control group). Baseline characteristics were balanced between antihypertensive treatment and control groups in participants with hypertension history and those without hypertension history (all *P* > .05; Table 1).

**Table 1.** Baseline Characteristics of Participants According to History of Hypertension Before Ischemic Stroke Onset

Characteristic	No. (%) of Patients					
	With Hypertension			Without Hypertension		
	Treatment (n = 1610)	Control (n = 1599)	*P* Value^a^	Treatment (n = 428)	Control (n = 434)	*P* Value^a^
Male	1014 (63.0)	1001 (62.6)	.83	303 (70.8)	286 (65.9)	.12
Age, mean (SD), y	62.1 (10.7)	61.5 (10.8)	.14	62.0 (11.0)	63.0 (11.5)	.19
Time from onset to randomization, mean (SD), h	15.7 (13.1)	15.0 (13.1)	.14	13.8 (11.9)	14.4 (12.6)	.47
Blood pressure at entry, mean (SD), mm Hg						
Systolic	166.9 (17.3)	165.9 (16.7)	.12	165.9 (17.0)	164.4 (15.7)	.17
Diastolic	97.0 (10.8)	96.8 (11.6)	.60	96.0 (11.0)	95.6 (10.3)	.55
BMI, mean (SD)	25.1 (3.2)	25.1 (3.1)	.50	24.4 (3.0)	24.5 (3.2)	.76
Hyperlipidemia	119 (7.4)	124 (7.8)	.70	18 (4.2)	16 (3.7)	.70
Diabetes	321 (19.9)	300 (18.8)	.40	48 (11.2)	50 (11.5)	.89
Coronary heart disease	189 (11.7)	192 (12.0)	.81	27 (6.3)	36 (8.3)	.26
Current cigarette smoking	535 (33.2)	565 (35.3)	.21	190 (44.4)	195 (44.9)	.87
Current alcohol consumption	441 (27.4)	484 (30.3)	.07	173 (40.4)	155 (35.7)	.15
NIH Stroke Scale score at baseline, median (IQR)^b^	4.0 (2.0-7.0)	4.0 (3.0-7.0)	.21	4.0 (2.0-8.0)	5.0 (3.0-8.0)	.23
Ischemic stroke subtype^c^						
Thrombotic	1255 (78.0)	1273 (79.6)	.25	320 (74.8)	322 (74.2)	.85
Embolic	81 (5.0)	75 (4.7)	.65	18 (4.2)	28 (6.5)	.14
Lacunar	316 (19.6)	296 (18.5)	.42	101 (23.6)	89 (20.5)	.27

Abbreviations: BMI, body mass index (calculated as weight in kilograms divided by height in meters squared); IQR, interquartile range; NIH, National Institutes of Health.

^a^ Difference between the antihypertensive treatment and control groups was tested for means using *t* tests, percentages using χ^2^ tests, and medians using Wilcoxon rank sum tests.

^b^ Scores range from 0 (normal neurologic status) to 42 (coma with quadriplegia).

^c^ For these subtypes, 12 patients had both thrombotic and embolic; 93 had thrombotic and lacunar; 6 had embolic and lacunar ischemic stroke; and 1 had all 3 subtypes.

### Blood Pressure Decrease

**Figure 2.**
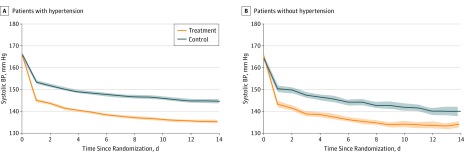
Systolic Blood Pressure (BP) Since Randomization by History of Hypertension A, Numbers at risk at entry were 1610 for the treatment group and 1599 for the control group. B, Numbers at risk at entry were 428 for the treatment group and 434 for the control group. Solid lines indicate mean BP; shading, 95% CI.

At 24 hours after randomization, the differences in systolic BP between the intervention group and control group were −8.4 (95% CI, −9.5 to −7.3) mm Hg (*P* < .001) in the patients with history of hypertension and −7.2 (95% CI, −9.3 to −5.1) mm Hg (*P* < .001) in those without history of hypertension (eTable 1 in Supplement 2). Within 24 hours after randomization, mean (SD) systolic BP was decreased by 21.7 (16.3) mm Hg (12.6%) in the early antihypertensive treatment group and by 12.4 (17.4) mm Hg (7.0%) in the control group for patients with a hypertension history. The mean (SD) systolic BP was decreased by 22.4 (14.5) mm Hg (13.2%) in the treatment group and 14.0 (17.1) mm Hg (8.1%) in the control group for patients without hypertension history (Figure 2; eTable 1 in Supplement 2). The corresponding systolic BP differences between the treatment and control groups were −9.3 (95% CI, −10.5 to −8.2) mm Hg for patients with a hypertension history and −8.4 (95% CI, −10.5 to −6.2) mm Hg for those without a hypertension history (both *P* < .001). The systolic BP differences between the treatment and control groups were −9.4 (95% CI, −10.3 to −8.5) mm Hg (*P* < .001) for patients with a hypertension history and −8.8 (95% CI, −10.6 to −7.0) mm Hg (*P* < .001) for those without a hypertension history at day 7 after randomization, and −9.3 (95% CI, −10.6 to −8.0) mm Hg for patients with a hypertension history and −5.8 (95% CI, −8.4 to −3.1) mm Hg for those without a hypertension history at day 14 after randomization (both *P* < .001).

### Clinical Outcomes at 14 Days or Hospital Discharge

At 14 days or hospital discharge, the primary outcome of death or major disability was not significantly different between the antihypertensive treatment among participants with a history of hypertension or among those without a history of hypertension (*P* = .97 for homogeneity). The ORs associated with early antihypertensive treatment were 1.00 (95% CI, 0.87-1.16) for participants with hypertension and 1.00 (95% CI, 0.75-1.32) for those without hypertension (Table 2). Likewise, the secondary outcomes of the mRS scores (*P* = .36 for homogeneity) and death (*P* = .98 for homogeneity) were not significantly different between the treatment and control groups.

**Table 2.** Clinical Outcomes at 14 Days or Hospital Discharge According to History of Hypertension Before Ischemic Stroke Onset

Outcome Variable	No. (%) of Patients								*P* Value for Homogeneity^a^
	With Hypertension				Without Hypertension				
	Treatment (n = 1603)	Control (n = 1597)	OR (95% CI)	*P* Value^a^	Treatment (n = 428)	Control (n = 430)	OR (95% CI)	*P* Value^a^	
Primary outcome									
Death or major disability^b^	533 (33.3)	530 (33.2)	1.00 (0.87-1.16)	.97	150 (35.0)	151 (35.1)	1.00 (0.75-1.32)	.98	.97
Secondary outcomes									
Modified Rankin Scale score, median (IQR)	2.0 (1.0-3.0)	2.0 (1.0-3.0)		.99	2.0 (1.0-3.0)	2.0 (1.0-3.0)		.38	.36
0 (No symptoms)	162 (10.1)	129 (8.1)	1.00 (0.89-1.13)		42 (9.8)	25 (5.8)	0.90 (0.71-1.14)		.36
1 (No significant disability despite symptoms)	519 (32.4)	555 (34.8)			134 (31.3)	146 (34.0)			
2 (Slight disability)	389 (24.3)	383 (24.0)			102 (23.8)	108 (25.1)			
3 (Moderate disability)	217 (13.5)	236 (14.8)			75 (17.5)	61 (14.2)			
4 (Moderately severe disability)	207 (12.9)	214 (13.4)			51 (11.9)	68 (15.8)			
5 (Severe disability)	87 (5.4)	58 (3.6)			21 (4.9)	19 (4.4)			
6 (Death)	22 (1.4)	22 (1.4)			3 (0.7)	3 (0.7)			
Death	22 (1.4)	22 (1.4)	0.99 (0.55-1.80)	.98	3 (0.7)	3 (0.7)	1.01 (0.20-5.05)	.99	.98
Duration of initial hospitalization, median (IQR), d	13.0 (9.0-14.0)	13.0 (9.0-14.0)		.66	12.0 (9.0-14.0)	12.0 (7.0-14.0)		.14	.34

Abbreviations: IQR, interquartile range; OR, odds ratio.

^a^ Difference in the percentages of composite death or major disability and all-cause mortality between the antihypertensive treatment and control groups was tested using a χ^2^ test, the medians of the modified Rankin Scale score and hospitalization using the Wilcoxon rank sum test, and ORs of ordinal-modified Rankin Scale scores using ordinal logistic regression.

^b^ Modified Rankin Scale score of 3 or greater.

### Clinical Outcomes at 3-Month Follow-up

At the 3-month posttreatment follow-up visit, mean systolic and diastolic BPs were significantly lower in the antihypertensive treatment group than in the control group; the systolic BP differences were −3.1 (95% CI, −3.9 to −2.2) mm Hg (*P* < .001) for those with hypertension history and −2.4 (95% CI, −4.1 to −0.7) mm Hg (*P* = .005) for those without hypertension history, and the diastolic BP differences were −1.3 (95% CI, −1.9 to −0.7) mm Hg (*P* < .001) for those with hypertension history and −1.9 (95% CI, −3.0 to −0.8) mm Hg (*P* = .001) for those without hypertension history (Table 3). The composite outcome of death or major disability, mRS score, and death were all not different between the treatment and control groups by history of hypertension (*P* > .05 for homogeneity). For example, the ORs of the composite outcome of death or major disability associated with early antihypertensive treatment were 0.98 (95% CI, 0.83-1.15) for participants with hypertension and 1.06 (95% CI, 0.77-1.44) for participants without hypertension.

**Table 3.** Clinical Outcomes at 3-Month Posttreatment Follow-up Visit According to History of Hypertension Before Ischemic Stroke Onset

Outcome Variable	No. (%) of Patients									*P* Value for Homogeneity
	With Hypertension					Without Hypertension				
	Treatment (n = 1568)	Control (n = 1562)	BP Difference or OR (95% CI)	*P* Value	Treatment (n = 420)		Control (n = 425)	BP Difference or OR (95% CI)	*P* Value	
BP 3 mo after randomization, mean (SD), mm Hg										
Systolic	139.6 (11.3)	142.6 (12.7)	−3.1 (−3.9 to −2.2)	<.001	138.2 (12.9)		140.6 (11.7)	−2.4 (−4.1 to −0.7)	.005	.50
Diastolic	86.3 (7.8)	87.6 (8.0)	−1.3 (−1.9 to −0.7)	<.001	84.7 (8.0)		86.7 (7.8)	−1.9 (−3.0 to −0.8)	.001	.31
Death or major disability^a^	393 (25.1)	398 (25.5)	0.98 (0.83 to 1.15)	.79	107 (25.5)		104 (24.5)	1.06 (0.77 to 1.44)	.74	.67
Modified Rankin Scale score, median (IQR)	1.0 (1.0 to 3.0)	1.0 (1.0 to 3.0)		.66	1.0 (1.0 to 3.0)		1.0 (1.0 to 2.0)		.57	.86
0 (No symptoms)	291 (18.6)	266 (17.0)	0.97 (0.86 to 1.10)^b^		86 (20.5)		75 (17.7)	0.93 (0.73 to 1.19)^b^		.86
1 (No significant disability despite symptoms)	527 (33.6)	545 (34.9)			139 (33.1)		145 (34.1)			
2 (Slight disability)	357 (22.8)	353 (22.6)			88 (21.0)		101 (23.8)			
3 (Moderate disability)	188 (12.0)	207 (13.3)			65 (15.5)		58 (13.7)			
4 (Moderately severe disability)	114 (7.3)	104 (6.7)			22 (5.2)		26 (6.1)			
5 (Severe disability)	39 (2.5)	42 (2.7)			4 (1.0)		11 (2.6)			
6 (Death)	52 (3.3)	45 (2.9)			16 (3.8)		9 (2.1)			
Death	52 (3.3)	45 (2.9)	1.16 (0.77 to 1.73)	.48	16 (3.8)		9 (2.1)	1.83 (0.80 to 4.19)	.15	.33
Recurrent stroke	18 (1.2)	40 (2.6)	0.44 (0.25 to 0.77)	.004	10 (2.4)		3 (0.7)	3.43 (0.94 to 12.55)	.06	.005
Vascular events^c^	35 (2.3)	52 (3.3)	0.66 (0.43 to 1.02)	.06	13 (3.1)		7 (1.7)	1.91 (0.75 to 4.83)	.17	.04

Abbreviations: BP, blood pressure; IQR, interquartile range; OR, odds ratio.

^a^ Modified Rankin score of 3 or greater.

^b^ Odds of a 1-unit higher modified Rankin Scale score.

^c^ Includes vascular deaths, nonfatal stroke, nonfatal myocardial infarction, hospitalized and treated angina, hospitalized and treated congestive heart failure, and hospitalized and treated peripheral arterial disease.

However, the early antihypertensive treatment effects on recurrent stroke (*P* = .005 for homogeneity) and vascular events (*P* = .04 for homogeneity) were statistically different between patients with or without history of hypertension. Antihypertensive treatment was associated with a 56% decreased risk of recurrent stroke (OR, 0.44; 95% CI, 0.25-0.77; *P* = .004) in patients with acute ischemic stroke and history of hypertension, whereas no such association was observed among patients without history of hypertension (OR, 3.43; 95% CI, 0.94-12.55; *P* = .06).

### Sensitivity Analysis

Further adjustment for age, sex, baseline systolic BP, and other potential covariates in the multivariable model did not significantly change the association between antihypertensive treatment and patient outcomes (eTable 2 in Supplement 2). With antihypertensive treatment, the ORs for the primary outcome at 14 days were 1.03 (95% CI, 0.84-1.26) for patients with history of hypertension and 1.19 (95% CI, 0.82-1.74) for patients without history of hypertension (*P* = .40 for homogeneity). The association between early antihypertensive treatment and 3-month recurrent stroke (*P* = .01 for homogeneity) and vascular events (*P* = .05 for homogeneity) were different between patients with or without history of hypertension.

## Discussion

In this prespecified subgroup analysis of the CATIS trial, we found that mean systolic BP was decreased more substantially in the intervention group than in the control group both in patients with or without history of hypertension at 14 days or hospital discharge and at the 3-month posttreatment follow-up after randomization. However, early antihypertensive intervention was not associated with the primary outcome of death or major disability at 14 days or hospital discharge or with the leading secondary outcome of death or major disability at 3 months both in patients with or without a history of hypertension. By contrast, immediate antihypertensive intervention was associated with a different likelihood of 3-month recurrent stroke and vascular events by hypertension history, with a significantly decreased risk of recurrent stroke events observed among patients with a history of hypertension treated with early antihypertensive therapy. Our study findings contribute to better BP management in the acute phase of ischemic stroke in several ways.

First, BP is recognized as a strongly predictive factor of the risks of both first and recurrent stroke,^12,21^ whereas there are limited data from clinical trials on the effect of antihypertensive treatment in patients with acute ischemic stroke, especially those with or without a history of hypertension. The CATIS trial found that immediate BP decrease within 48 hours after symptom onset did not decrease the likelihood of death or dependency among patients with acute ischemic stroke.^14^ The present subgroup analysis confirmed the neutral effects of early antihypertensive treatment on death or major disability regardless of history of hypertension. However, our findings are also consistent with prior clinical trials in which antihypertensive intervention had similar effects on functional outcome in patients with or without history of hypertension.^15,16^ The Efficacy of Nitric Oxide in Stroke (ENOS) trial recruited 4011 patients with acute ischemic or hemorrhagic stroke and found that decreasing BP does not improve 6-month functional outcome and that there is no significant interaction between antihypertensive treatment and history of hypertension among patients with acute stroke.^16^ Notably, our study included only patients with ischemic stroke and provided an opportunity to assess the potential modification effect of history of hypertension on the relationship between early BP decrease and clinical outcomes specifically in patients with ischemic stroke.

Second, decreasing BP has been established as an effective treatment for the prevention of first stroke among individuals with hypertension.^22,23^ Furthermore, the Perindopril Protection Against Recurrent Stroke Study (PROGRESS) reported a beneficial effect of decreasing BP in the secondary prevention of stroke among individuals with hypertension and a history of stroke or transient ischemic attack within the previous 5 years.^24^ Our findings extended this information specifically to the acute phase of ischemic stroke. Early antihypertensive intervention in the present study was associated with a 56% decrease in recurrent stroke and a 34% decrease in vascular events in ischemic stroke with hypertension. However, the Scandinavian Candesartan Acute Stroke Trial (SCAST) reported no evidence of a beneficial effect of a BP-decreasing treatment with an angiotensin-receptor blocker in patients with acute stroke and raised BP.^17^ Several potential reasons might account for the inconsistent findings. One is that the early absolute difference in systolic BP between the intervention group and control group 24 hours after randomization (8.4 mm Hg in the patients with history of hypertension and 7.2 mm Hg in those without history of hypertension) was substantially greater than that in SCAST study (0.4 mm Hg); this larger achieved BP reduction in the CATIS trial enable us to detect the protective effect of decreasing BP on recurrent stroke. Another possible explanation is that the SCAST study included both patients with acute ischemia and those with hemorrhagic stroke, and the different pathophysiological mechanisms may have caused the inconsistent results between the studies.

Third, in the PROGRESS trial, a beneficial effect of decreasing BP was also observed in nonhypertensive individuals with prior cerebrovascular disease.^24^ However, we did not observe any protective effects of decreasing BP in the acute phase on death, recurrent stroke, and vascular events in patients without history of hypertension. Early increased BP is caused in part by a response to physical and psychological stresses from brain ischemia, and this transient increase in BP would spontaneously decline by about one-quarter in most patients within the first 24 hours after stroke onset.^10,11^ Early BP decrease may reduce collateral flow through arteries and then increase the size of the cerebral infarction, which may further lead to recurrent stroke and other adverse events, especially for hypoperfused or patients without hypertension before stroke onset. We hypothesized that in patients without hypertension, BP-decreasing treatment is more likely to lead to hypoperfusion and an increase in infarct, which may offset the potential beneficial effects of treatment on reducing vascular damage, cerebral edema, and hemorrhagic transformation.

Our findings have important clinical implications. This subgroup analysis provides data to support early antihypertensive intervention among patients with ischemic stroke and a history of hypertension, and early treatment could help them transition to long-term antihypertensive therapy for secondary prevention; for patients without prior hypertension, the decision to decrease BP with antihypertensive treatment should be based on individual clinical judgment and requires more caution. Time from stroke onset to initiation of antihypertensive treatment has been identified as a potential effect modification of the early antihypertensive intervention on clinical outcomes.^25,26^ Apart from the duration of initial hospitalization, history of hypertension should also be considered in deciding whether to decrease BP or not in the acute phase of ischemic stroke.

### Limitations

Our findings should be interpreted in light of some limitations. First, as a subgroup analysis of a clinical trial, the statistical power might be decreased because of some methodological issues, such as multiple comparisons.^27^ Therefore, chance or confounding factors could be associated with our findings, and we could not provide a definite answer to guide clinical patient care. However, the study findings did not significantly change after adjusting for important associated factors. Second, data regarding cerebral blood flow, collateral blood flow, and presence of penumbral tissue were not collected in the CATIS trial, which may limit more precise estimates of early antihypertensive intervention in the present study. Third, history of hypertension was defined as *yes* answers to 2 questions about previous diagnoses and current use of antihypertensive medications; thus, this self-reported hypertension might not include all patients with hypertension prior to trial enrollment and might cause misclassification of several hypertensive patients. Fourth, our study participants were exclusively Chinese patients with stroke, and the findings from our study might not be generalizable to other populations in whom the management of acute ischemic stroke might be different.

## Conclusions

Our prespecified subgroup analysis of the CATIS trial indicated that early antihypertensive treatment was not associated with death and major disability among patients with ischemic stroke with or without hypertension. However, early antihypertensive therapy was associated with lower rates of 3-month recurrent stroke among patients with history of hypertension. Future clinical trials are warranted to confirm our findings.

## Notes

Supplement 1. **Trial Protocol**

Supplement 2. **eTable 1.** Blood Pressure Reduction After Randomization at 14 Days or Hospital Discharge According to History of Hypertension **eTable 2.** Adjusted Odds Ratios (95% Confidence Intervals) According to History of Hypertension Before Ischemic Stroke Onset **eFigure.** Treatment Algorithm for Blood Pressure Reduction Group
