# Supplementary material for: Immediate Antihypertensive Treatment for Patients With Acute Ischemic Stroke With or Without History of Hypertension: A Secondary Analysis of the CATIS Randomized Clinical Trial
Source: JAMA Netw Open. 2019 Jul 31;2(7):e198103. doi: 10.1001/jamanetworkopen.2019.8103 (PMC6669782; doi:10.1001/jamanetworkopen.2019.8103)
Supplement: Supplement 1. — Trial Protocol [file jamanetwopen-2-e198103-s001.pdf]

CHINA ANTIHYPERTENSIVE TRIAL IN ACUTE ISCHEMIC STROKE (CATIS)

STUDY PROTOCOL

CATIS Study Steering Committee

Revised:

September 2007
April 2009
August 2009
April 2013

Incorporating Amendment #1, Dated: October 2009
Incorporating Amendment #2, Dated: November 2009
Incorporating Amendment #3, Dated: August 2011

New Orleans, USA – Suzhou, China

China Antihypertensive Trial in Acute Ischemic Stroke (CATIS)

Study Protocol

1. BACKROUND

Stroke is a major cause of death and disability in the Chinese population, and the incidence and mortality of stroke is generally much higher than in western populations (1-3). Hypertension is the most important modifiable risk factor for stroke (4). The association between blood pressure and risk of stroke has been investigated in many prospective observational studies. Results from these prospective studies have been combined in several major pooling projects that have demonstrated that blood pressure is positively, continuously, and independently associated with an increased risk of stroke (5-7). In the Eastern Stroke and Coronary Heart Disease Collaborative Research, which included 13 cohorts from China and 5 from Japan (124,774 participants, 837,214 person-years of observation), each 5 mm Hg lower usual diastolic blood pressure was associated with lower risk of non-hemorrhagic stroke (odds ratio 0.61 [95% CI 0.57-0.66]) and lower risk of hemorrhagic stroke (0.54 [0.50-0.58]) (7).

Over the past several decades, many antihypertensive drug treatment trials have been conducted to determine whether blood pressure reduction decreases the risk of stroke among hypertensive patients or stroke survivors (8-10). Results from these trials have been pooled in several meta-analyses to obtain a more precise and accurate estimate of the effect of antihypertensive treatment on clinical outcomes (8,9). These meta-analyses indicated that anti-hypertensive treatment was associated with a 38% reduction in stroke incidence and a 40% reduction in stroke mortality (8,9). In the Perindopril Protection Against Recurrent Stroke Study (PROGRESS) trial, 6,105 hypertensive and non-hypertensive patients with a history of stroke or transient ischemic attack were randomly assigned active treatment (n=3,051) or placebo (n=3,054) (10). Active treatment comprised a flexible regimen based on the angiotensin-converting-enzyme inhibitor perindopril (4 mg daily) with the addition of the diuretic indapamide at the discretion of treating physicians. Over 4 years of follow up, active treatment reduced blood pressure by 9/4 mm Hg. Ten percent (n=307) of individuals assigned to active treatment suffered a stroke, compared with 14% (n=420) assigned placebo (relative risk reduction 28% [95% CI 17–38%], $p<0.0001$). There were similar reductions in the risk of stroke in hypertensive and non-hypertensive subgroups (all $p<0.01$). Combination therapy with

perindopril plus indapamide reduced blood pressure by 12/5 mm Hg and stroke risk by 43% (95% CI 30-54%). This trial indicated that lowering blood pressure reduced the risk of stroke among both hypertensive and non-hypertensive individuals with a history of stroke or transient ischemic attack (10).

However, the effect of blood pressure reduction among acute stroke patients is much less certain (11,12). There is no evidence from randomized clinical trials to guide clinical decisions on blood pressure management. For many years, there has been a debate about whether to lower or to raise blood pressure and whether to continue or to stop antihypertensive treatment among acute stroke patients (12). This situation is reflected in the inconsistent and conflicting advice given in national and international guidelines on blood pressure management among acute stroke patients (13-18). Indeed, the lack of evidence helps to explain why guidelines and textbooks barely mention blood pressure in the management of acute stroke, and when they do, their recommendations are cautious and conservative.

The European Stroke Initiative recommends initiation of blood pressure-lowering therapy for acute stroke when blood pressure is greater than 220/120 mm Hg, with a target of 180/100-105 mm Hg for known hypertensive patients and 160-180/90-105 mm Hg for non-hypertensive patients (14). The American Stroke Association similarly recommends initiation of antihypertensive therapy when blood pressure is greater than 200/120 mm Hg for acute ischemic stroke without thrombolysis, but the threshold is lower for starting antihypertensive therapy during and after thrombolysis - when blood pressure is greater than 180/105 mm Hg (14). Few recommendations provide definitive advice on initiating antihypertensive therapy among acute stroke patients with blood pressures <220/120 mm Hg. Thus, the most recent Cochrane Collaboration Stroke Review concludes that it is not clear whether high blood pressure should be lowered in acute stroke, or whether antihypertensive drugs should be continued or stopped (13).

The World Health Organization-International Society of Hypertension issued a "Statement on the Management of Blood Pressure in Acute Stroke," which underlined the confused state of knowledge and the need for new evidence in this area (16). The statement advocated further research on four major issues: (i) should blood pressure be lowered in acute ischemic stroke; (ii) should blood pressure be elevated in acute ischemic stroke when there is evidence of hypoperfusion; (iii) should blood pressure be lowered in primary intracerebral

hemorrhage; and (iv) should previous antihypertensive therapy be continued or stopped temporarily?

The Acute Candesartan Cilexetil Therapy in Stroke Survivors (ACCESS) study was designed to assess the safety of modest blood pressure reduction by candesartan cilexetil among acute stroke patients with blood pressure ≥200/110 mm Hg (19). Among 342 acute stroke patients (339 confirmed) who were randomized to treatment with angiotensin type-1 receptor blockade or placebo control, the cumulative 12-month mortality and the number of vascular events differed significantly in favor of the candesartan cilexetil group (odds ratio, 0.48; 95% CI, 0.25 to 0.90). However, blood pressure on admission, on study onset, and throughout the whole study period was not significantly different between the two groups (19).

There are two on-going clinical trials designed to examine stopping antihypertensive medications among acute stroke patients (20-22). The Efficacy of Nitric Oxide in Stroke (ENOS) study is an international, multi-center, randomized, blinded, controlled trial designed to test the safety and efficacy of transdermal glyceryl trinitrate (releasing nitric oxide, a vasodilator) or control and of continuing or temporarily stopping prior antihypertensive medication among 5,000 patients with acute ischemic or hemorrhagic stroke within 48 hours of the onset of stroke. Treatment will be given for 7 days. The primary outcome is death or dependency (modified Rankin scale >2) and will be determined at 3 months by observers blinded to treatment (20). Continue or Stop Post-Stroke Antihypertensive Collaborative Study (COSSACS) is a multi-center, prospective, randomized, open, blinded end-point study (PROBE design) that will randomize 2900 patients who are in the acute phase of stroke, and who are on pre-existing antihypertensive therapy, to continue or stop current antihypertensive medications (21). Eligible patients must be admitted to the hospital within 24 hours of the onset of the suspected stroke and within 36 hours of the last dose of antihypertensive medication. The primary outcome will be the proportion of patients who are dead or dependent (defined as a modified Rankin score >2) at 14 days post-stroke, and secondary outcomes include blood pressure changes and functional status at 2 weeks and 6 months post-stroke (21).

The Controlling Hypertension and Hypotension Immediately Post-Stroke (CHHIPS) Pilot Trial is a UK based multi-center, randomized, double-blind, placebo-controlled, titrated dose trial designed to assess whether hypertension and relative hypotension, manipulated therapeutically in the first 24 hours following acute stroke, affects short-term outcome measures

(22). The CHHIPS Pilot Study aims to recruit 2,050 patients with clinically suspected stroke confirmed by brain imaging who have no compelling indication or contraindication for blood pressure manipulation. The primary outcome measure will be the effects of acute pressor therapy (initiated ≤12 hours from stroke onset) or depressor therapy (started ≤24 hours post-ictus) on death and dependency at 14 days post-stroke. These trials, however, do not address the question of whether blood-pressure-lowering reduces case-fatality and disability among patients with acute stroke.

2. OBJECTIVES

The China Antihypertensive Trial in Acute Ischemic Stroke (CATIS) is designed to determine whether lowering systolic blood pressure by 10-25% within the first 24 hours and achieving systolic blood pressure <140 and diastolic blood pressure <90 mm Hg within 7 days after randomization and maintaining this blood pressure level afterwards during hospitalization in acute ischemic stroke will reduce short-term case-fatality and major disability within 2-weeks and long-term mortality, major disability, stroke recurrence, and vascular events over 3, 12, and 24 months as compared to usual care based on current clinical guidelines.

The primary objective of the CATIS is to test the effect of early blood pressure reduction on short-term case-fatality and major disability (modified Rankin scale ≥3) among patients with acute ischemic stroke within 2 weeks.

The secondary objective of the CATIS is to test the effect of early blood pressure reduction on long-term mortality and major disability among patients with acute ischemic stroke over 3, 12, and 24 months.

The other secondary objectives of the CATIS are to test the effect of blood pressure reduction among patients with acute ischemic stroke on the following outcomes over 3, 12, and 24 months:

- Recurrent stroke
- Vascular events
- All-cause mortality
- Combination of vascular events and all cause-mortality
- Neurological functional status

We will collect DNA and serum/plasma specimens for future genomics and clinical studies on the etiology and prognosis of ischemic stroke.

3. STUDY DESIGN AND POWER

3.1. Study Design

The proposed study is a multi-center, randomized, controlled trial with two parallel arms:

- Active intervention arm: Initial antihypertensive treatment with angiotensin-converting enzyme inhibitors (Enalapril) and/or calcium channel blockers as second line medication; and/or diuretics as third line medications. Based on patients' baseline blood pressure levels, the first-line medication (intravenous Enalapril) can be used alone, or in combination with second-line medication (calcium channel blocker), and third-line medication (diuretics) to achieve the target systolic blood pressure lowering by 10% to 25% within the first 24 hours after randomization (see Appendix A) and to achieve systolic blood pressure below 140 mm Hg and diastolic blood pressure below 90 mm Hg and maintain this blood pressure level afterwards during the hospitalization.
- Usual care arm: Discontinue all home blood pressure medications.

Besides receiving or not receiving antihypertensive treatment, both groups will receive standard care according to the China National Guidelines for Prevention and Treatment of Cerebrovascular Diseases. After discharge, all study participants will receive standard care provided by their own healthcare providers according to the national guidelines. Antihypertensive medications will be prescribed to all study participants by their physicians.

3.2. Blinding

In this trial, the patients and study investigators (and staff) who collect the outcome data will be blinded to the patient's randomization assignment. The Outcome Assessment Committee will also be blinded to the patient's randomization assignment. However, the study physicians who are treating study participants will not be blinded.

3.3 Sample Size and Statistical Power

The sample size calculation is based on the primary outcome (combination of death within 14 days after randomization and major disability at 14 days or at the time of discharge, if that occurred before 14 days). We calculated sample size based on the following assumptions:

- Significance level of 0.05 for a two-sided test;

- Statistical power of 90%;
- Event rate in control group of 35% based on data from the CATIS-vanguard phase, which is more conservative than those reported from previous studies (23);
- Absolute risk reduction of 5%, which has been recommended as clinically meaningful risk reduction in acute stroke trials (24).

We estimated that 1,842 patients are required for each group based on a likelihood ratio test, and we will recruit 4,000 patients (2,000 for each group) in the CATIS trial to accommodate potential withdrawals. In addition, we have 80% statistical power to detect a 20% proportional reduction in the combined outcome of mortality and dependency over 3 months of follow-up (23.4% in control group from the CATIS-vanguard phase) based on a two-sided likelihood ratio test at the significance level of 0.05.

4. ELIGIBILITY AND EXCLUSION CRITERIA

This randomized clinical trial will only include patients with acute ischemic stroke. Ischemic stroke includes:

- Thrombotic stroke
- Embolic stroke
- Lacunar cerebral infarction
- Other types of ischemic stroke.

The eligibility criteria for participation are:

Inclusion Criteria

- Age ≥22 years
- Ischemic stroke onset within 48 hours confirmed by imaging (CT scan or MRI) study
- Systolic blood pressure ≥140 and <220 mm Hg and diastolic blood pressure ≥80 mm Hg
- No contraindications to antihypertensive treatment
- Able and willing to sign informed consent by patients or their direct family members

Exclusion criteria

- Hemorrhagic stroke

- Severe heart failure (NY Heart Association class III and IV), myocardial infarction, unstable angina, atrial fibrillation, aortic dissection and cerebrovascular stenosis (>70%)
- Patients in a deep coma
- Diastolic blood pressure >120 mm Hg
- Resistant hypertension (systolic blood pressure ≥170 mm Hg despite use of ≥4 antihypertensive medications for ≥6 months)
- Intravenous thrombolytic therapy (such as intravenous rtPA)
- Unable to participate in the follow-up examination (i.e., living more than 30 kilometers away from participating hospital)

5. RECRUITMENT AND RANDOMIZATION

We will recruit 4,000 acute ischemic stroke patients from 26 hospitals in 7 regions in China (Hebei, Heilongjiang, Inner Mongolia, Jiangsu, Jiangxi, Jilin, and Shandong provinces). The recruitment period will last for 44 months. The targeted recruitments by region are:

- Hebei, 5 hospitals
- Heilongjiang, 2 hospitals
- Inner Mongolia, 4 hospitals
- Jiangsu, 3 hospitals
- Jilin, 4 hospitals
- Liaoning, 4 hospitals
- Shandong, 4 hospitals

The study coordinators or study physicians at each hospital will approach all acute stroke patients as soon as allowed based on the patient's clinical condition (within 6 hours of hospitalization and within 48 hours of disease onset). Three blood pressure measures will be obtained using a standard mercury sphygmomanometer, and the mean of the three blood pressure measures will be used to determine eligibility. Other criteria will be assessed by study questionnaire or by clinical examination.

Randomization will be conducted centrally at the Data Coordinating Center. Randomization will be stratified by hospitals and history of antihypertensive treatment. In each stratum, patients will be randomly assigned to the blood pressure lowering or the control group within each block. The block size will be random among 4, 6, and 8. The intervention group will receive the antihypertensive treatment according to study protocol, and the control group will receive usual medical care provided by local hospitals and will not receive antihypertensive treatment except under circumstances of safety concern (see **Safety Consideration** section).

6. ANTIHYPERTENSIVE INTERVENTION

After the study participants are randomly assigned into the active antihypertensive treatment group and the usual care control group, all home blood pressure medications will be discontinued. The active treatment group will receive antihypertensive medications according to the study protocol and the control group will receive standard care according to *the China National Guidelines for Prevention and Treatment of Cerebrovascular Diseases* without antihypertensive treatment during the hospitalization (approximately 2-weeks).

6.1. Antihypertensive Agents

For the intervention group, antihypertensive agents include intravenous angiotensin-converting enzyme inhibitors (Enalapril, first-line), calcium channel blockers (second-line) and diuretics (third-line). The primary objective of this clinical trial is to test an antihypertensive strategy not specific antihypertensive medications. Therefore, study physicians can select calcium channel blockers and diuretics based on the availability of these medications in their hospitals.

6.2 Treatment Plan

A. Blood Pressure Goals and Timeline:

Blood pressure-lowering will start immediately after randomization. The targeting treatment goals are:

- Step 1 (within 24 hours after randomization)

To lower systolic blood pressure by 10 to 25% (but systolic blood pressure not lower than 126 mm Hg and diastolic blood pressure not lower than 80 mm Hg) within the first 24 hours after randomization based on the participant’s admission blood pressure levels

- Step 2 (within 7 days after randomization)

To achieve systolic blood pressure below 140 mm Hg and diastolic blood pressure below 90 mm Hg and maintain this blood pressure level afterwards

Except for the antihypertensive intervention, the intervention group will receive the same usual care as the control group according to the China National Guidelines for Prevention and Treatment of Cerebrovascular Diseases.

B. Medication Administration:

All participants in the intervention group will receive antihypertensive medications in a staged sequence (see Appendix A). Based on patients' baseline blood pressure level, the first-line medication (intravenous angiotensin-converting enzyme inhibitor) can be used alone, or in combination with second-line medication (calcium channel blocker), and third-line medication (diuretics) to achieve the target systolic blood pressure lowering by 10 to 25% within the first 24 hours after randomization. The dosage and number of medications will be titrated to achieve systolic blood pressure below 140 mm Hg and diastolic blood pressure below 90 mm Hg within 7 days and maintain this blood pressure level afterwards.

6.3. Safety Consideration

- Physicians will closely monitor stroke patients during the intervention. Patients who experience severe conditions during the intervention, such as neurologic status worsened, acute myocardial infarction, and heart failure, will be withdrawn from the trial.
- Stroke patients with mean systolic blood pressure ≥220 or diastolic blood pressure ≥120 mm Hg during intervention will be withdrawn from the trial.
- Physicians will collect endpoint data for all withdrawn patients. These data will be used in the final statistical analysis according to the intention-to-treat principle.
- A Data and Safety Monitoring Board will examine data every 6-12 months. If unexpected and highly significantly ($p<0.0001$) beneficial or harmful effects are identified, the DSMB could recommend stopping the trial. However, the DSMB decision should not only depend on the statistical analysis. The overall benefits and harms to the study participants and the importance of the knowledge to be gained should be balanced.

6.4. Data and Safety Monitoring Plan

An independent Data and Safety Monitoring Board (DSMB) will oversee the protections of human subjects from research (see Appendix B). The DSMB has reviewed and approved this study protocol. The study protocol has also been approved by the Tulane University Institutional

Review Board (IRB) in the US and Soochow University Research Ethics Committee in China. The investigators will report any unexpected adverse events to the DSMB and IRB in a timely fashion. The DSMB will review study process and safety issues every 6-12 months.

Written informed consent, which has been approved by the Tulane University IRB in the US and Soochow University Research Ethics Committee in China, will be obtained from all study participants. If study participants are capable of communicating with study staff, we will require them to sign the consent from. Otherwise, we will obtain written consent from their family members in situations such as patients in coma or unable to communicate.

7. STUDY VISIT AND DATA COLLECTION

7.1. Baseline Examination

Informed consent will be obtained at the baseline (BL)/randomization visit (Table 1). Information on medical history and antihypertensive medication will be collected. A CT/MRI test will be conducted to confirm the diagnosis of ischemic stroke. Three brachial blood pressure measurements will be taken 30 seconds apart at the baseline examination. Neurological and functional assessments including the modified Rankin scale and the NIH Stroke Scale (NIHSS) will be performed.

7.2. Trial Follow-up Examinations

Blood pressure will be measured every two hours during the first 24 hours, every four hours between 24 and 72 hours, and three times daily during days 4 to 14. At each blood pressure assessment, three brachial blood pressure measurements will be taken 30 seconds apart using a standard mercury sphygmomanometer. A CT or MRI test will be conducted on day 7 after the randomization to assess the progress of ischemic stroke. Neurological and functional assessments including the modified Rankin scale and NIHSS will be obtained 14 days after randomization or at the time of discharge, if that occurred before 14 days. ***All study outcomes will be measured by an observer who is blinded to the patient's randomization assignment***.

7.3. Post-trial Follow-up Examinations

Those patients who are still alive at hospital discharge will be contacted by telephone to set up a follow-up clinical visit. Information on clinical vascular events and deaths will be obtained. Current treatment, including antihypertensive therapy, will also be recorded. Neurological and functional assessments including the modified Rankin scale and NIHSS will be

performed. In addition, information on quality of life and cognitive function (24,25) will be obtained. ***Long-term follow-up at 3, 12 and 24 months will be conducted by an observer who is blinded to the patient's randomization assignment.***

A study outcome assessment committee who are blinded to the patient's randomization assignment will review all short-term outcomes (14-day death and functional status) and long-term outcomes (vascular events, death, and functional status). The adjudication of vascular events will be based on criteria established in the ALLHAT trial.

Table 1. Trial visiting schedule and data collection

	BL	Intervention, days				Post-trial follow-up, months		
		1	2-3	4-13	14	3	12	24
Informed consent	√							
Medical history	√				√	√	√	√
CT/MRI	√			√*				
Blood pressure	3	3/2 hr	3/4 hr	3x3/day	3	3	3	3
Rankin scale and NIHSS	√				√	√	√	√
Clinical examination	√				√	√	√	√
QOL and cognitive function						√	√	√

* CT/MRI test on day 7.

8. OUTCOME MEASUREMENT

8.1. Outcomes within 2 weeks

- The primary outcome will be a combination of death within 14 days after randomization and major disability at 14 days or at the time of discharge, if that occurred before 14 days.
- The secondary outcome will be neurological dysfunction (modified Rankin scale and NIH Stroke Scale, NIHSS). Since the development of the initial CATIS protocol, ordinal analysis of modified Rankin scores has been recommended for acute stroke trials. Therefore, we will analyze the ordered 7-level categorical score of the modified Rankin scale as an outcome of neurological functional status (26,27).
- Additional secondary outcome is blood pressure changes during 14 days in hospital.

8.2. Outcomes in 3, 12, and 24 months

- The primary outcome will be a combination of all-cause mortality and major disability at 3, 12, and 24 months.
- The secondary outcomes will include the following variables at 3, 12, and 24 months:

– All-cause mortality
– Recurrent fatal and non-fatal stroke
– Combined vascular disease events (vascular deaths, non-fatal stroke, non-fatal myocardial infarction, coronary revascularization, hospitalized or treated angina, hospitalized or treated congestive heart failure, and hospitalized or treated peripheral arterial disease)
– Neurological functional status measured by NIHSS and modified Rankin scale
– Systolic and diastolic blood pressure at 3, 12, and 24 months

Modified Rankin Scale (28-30)

Score	Description
0	No symptoms at all
1	No significant disability despite symptoms; able to carry out all usual duties and activities
2	Slight disability; unable to carry out all previous activities, but able to look after own affairs without assistance
3	Moderate disability; requiring some help, but able to walk without assistance
4	Moderately severe disability; unable to walk without assistance and unable to attend to own bodily needs without assistance
5	Severe disability; bedridden, incontinent and requiring constant nursing care and attention
6	Dead

- An ancillary outcome will be cognitive impairment at 3, 12, and 24 months in a subgroup of patients. The modified Mini-Mental State Examination (MMSE) and the Montreal Cognitive Assessment (MoCA) will be used. The MMSE contains 20 items that measure the degree of cognitive impairment in the areas of orientation, concentration, language, praxis, and immediate and delayed memory (31,32). The MMSE showed high levels of sensitivity for moderate-to-severe cognitive impairment but lower levels for mild degrees of impairment (33), whereas MoCA, a brief 30-point global cognitive screen tool is more sensitive to mild cognitive impairment (34,35). Both MMSE and MoCA have been translated into Chinese and validated in the Chinese population (36,37).

9. QUALITY ASSURANCE AND QUALITY CONTROL

All study staff will be trained/certified and recertified throughout the trial on the study outcome assessments, including blood pressure measurements, neurological dysfunction assessments (NIHSS and modified Rankin scale), and clinical data collection. At the training sessions, interviewers will be given detailed instructions on administration of the study questionnaire. All blood pressure observers will participate in a special training session on the use of a standardized protocol for measurement of blood pressure. Satisfactory performance during a written test on knowledge of preparing study participants for blood pressure measurement, selecting correct cuff size and using standard techniques for blood pressure measurement during a standardized videotape examination and during concordant measurements of blood pressure with an instructor will be required for certification as a blood pressure observer.

All data will be double entered through a web-based data system. The study data will be reviewed on a regular basis to identify incompleteness or errors by staff at the coordinating center. This information will be sent to the clinical center immediately for correction.

10. DATA MANAGEMENT AND ANALYSES

All data will be first entered at local hospitals and then sent to the Soochow University Department of Epidemiology where the second entry will be conducted. The two independent databases will be sent to Tulane University's Department of Epidemiology for final data check and quality control.

Data will be analyzed according to participants' randomized treatment assignments regardless of their subsequent medications (intent-to-treat analysis). Two-sided P-values will be calculated and the significance level will be assessed at 0.05. No imputation will be used for missing data. The proportions of participants with the primary and secondary outcomes at 14 days or discharge and at 3 months will be compared between the treatment and control groups using a χ^2 test. Logistic regression analysis will be used to estimate the unadjusted odds ratios and 95% confidence intervals associated with blood pressure reduction compared to usual control. In a sensitivity analysis, odds ratios will be adjusted for important confounders (baseline age, gender, systolic blood pressure, NIHSS score, time from onset of stroke to randomization, and use of antihypertensive medication). The median and inter-quartile range of Rankin's scale scores will be calculated for the two comparison groups, and the difference will be tested using a

Wilcoxon rank-sum test. Ordinal logistical regression will be used to estimate the effect of blood pressure reduction in the full range of the modified Rankin scale (31).

Cumulative event rates at 12 and 24 months will be calculated using the Kaplan-Meier method and differences will be tested using the log-rank test (32). A Cox proportional hazards regression model will be used to evaluate differences between cumulative event curves and adjust for important co-variables (baseline age, gender, systolic blood pressure, NIHSS score, time from onset of stroke to randomization, and use of antihypertensive medication). Hazard ratios (relative risks [RRs]) and 95% confidence intervals (CIs) will be obtained from the Cox proportional hazards regression model (32). The Cox proportional hazards regression model assumption will be examined using log-log plots and testing a treatment × time (time-dependent) interaction term; if it is violated, the RR estimate from a 2-by-2 table will be used (32).

Heterogeneity of effects in pre-specified subgroups, by age, gender, systolic blood pressure at baseline, NIHSS score at baseline, time from onset of stroke to randomization, history of hypertension, history of antihypertensive treatment, and subtypes of ischemic stroke will be examined by testing for treatment-covariate interaction with the logistic regression analysis or Cox proportional hazards regression model by using $P < 0.05$. SAS program (SAS Institute, Cary, NC) and STATA program (Stata Corp, College Station, TX) will be used for statistical analyses.

REFERENCES

1. WHO. Global Burden of Disease 2002: Deaths by age, sex and cause for the year 2002. Geneva, Switzerland: World Health Organization; 2003.

2. Yusuf S, Reddy S, Ounpuu S, Anand S. Global burden of cardiovascular diseases: Part II: variations in cardiovascular disease by specific ethnic groups and geographic regions and prevention strategies. Circulation. 2001;104:2855-2864.

3. He J, Gu D, Wu X, et al. Major causes of death among men and women in China. N Engl J Med. 2005;353:1124-1134.

4. The European Stroke Organisation (ESO) Executive Committee and the ESO Writing Committee. Guidelines for Management of Ischaemic Stroke and Transient Ischaemic Attack 2008. Cerebrovasc Dis. 2008;25:457-507.

5. MacMahon S, Peto R, Cutler J, et al. Blood pressure, stroke, and coronary heart disease, I: prolonged differences in blood pressure: prospective observational studies corrected for the regression dilution bias. Lancet 1990;335:765-74.

6. Prospective Studies Collaboration. Cholesterol, diastolic blood pressure, and stroke: 13,000 strokes in 450,000 people in 45 prospective cohorts. Lancet 1995;346:1647-53.

7. Eastern Stroke and Coronary Heart Disease Collaborative Research Group. Blood pressure, cholesterol, and stroke in eastern Asia. Lancet. 1998; 352:1801-7.

8. Collins R, Peto R, MacMahon SW, et al. Blood pressure, stroke, and CHD, II: short-term reductions in blood pressure: overview of randomized drug trials in their epidemiological context. Lancet 1990;335:827-38.

9. Whelton PK, He J. Blood pressure reduction. In: Manson JE, Buring JE, Ridker PM, Gaziano JM editors. Clinical trials in cardiovascular disease. 2nd ed. Philadelphia: Elsevier Saunders; 1999. p. 282-296.

10. PROGRESS Collaborative Group. Randomised trial of a perindopril-based blood-presure lowering regimen among 6105 individuals with previous stroke or transient ischaemic attack. Lancet. 2001; 358: 1033-1041.

11. Bath P, Chalmers J, Powers W, Beilin L, Davis S, Lenfant C, Mancia G, Neal B, Whitworth J, Zanchetti A. International Society of Hypertension Writing Group. International Society of Hypertension (ISH): statement on the management of blood pressure in acute stroke. J Hypertens. 2003; 21:665-72.

12. Chalmers J. Blood pressure in acute stroke: in search of evidence. J Hypertens. 2005; 23:277-8.

13. Blood Pressure in Acute Stroke Collaboration (BASC). Interventions for deliberately altering blood pressure in acute stroke (Cochrane Review). The Cochrane Library, issue 1. Chichester, UK: John Wiley & Sons; 2004.

14. Hacke W, Kaste M, Bogousslavsky J, Brainin M, Chamorro A, Lees K, et al, for the European Stroke Initiative Executive Committee. European stroke initiative recommendations for stroke management-update 2003. Cerebrovasc Dis 2003; 16:311-337.

15. Adams H, Adams R, Del Zoppo G, Goldstein LB. Guidelines for the early management of patients with ischemic stroke 2005 guideline update: A scientific statement from the Stroke Council of the American Heart Association/American Stroke Association. Stroke 2005; 36:916-921.

16. Bath P, Chalmers J, Powers W, Beilin L, Davis S, Lenfant C, et al, for the International Society of Hypertension Writing Group. International society of hypertension (ISH): Statement on the management of blood pressure in acute stroke. J Hypertens 2003; 21:665-672.

17. Lindley RI, Amayo EO, Marshall J, Sandercock PA, Dennis M, Warlow CP. Acute stroke treatment in UK hospitals: the Stroke Association survey of consultant opinion. JR Coll Physicians Lond 1995; 29:479-484.

18. Goldstein LB. Blood pressure management in patients with acute ischemic stroke. Hypertension 2004; 43:137-141.

19. Schrader J, Luders S, Kulschewski A, Berger J, Zidek W, Treib J, Einhaupl K, Diener HC, Dominiak P. The ACCESS study: evaluation of acute candesartan cilexetil therapy in stroke survivors. Stroke. 2003; 34: 1699-1703.

20. Efficacy of Nitric Oxide in Stroke trial. Available at: http://www.enos.ac.uk/

21. COSSACS Trial Group. COSSACS (Continue or Stop post-Stroke Antihypertensives Collaborative Study): rationale and design. J Hypertens. 2005; 23:455-8.

22. The CHHIPS Trial Group. CHHIPS (Controlling Hypertension and Hypotension Immediately Post-Stroke) Pilot Trial: rationale and design. J Hypertens. 2005; 23:649-55.

23. CAST (Chinese Acute Stroke Trial) Collaborative Group. CAST: randomised placebo-controlled trial of early aspirin use in 20,000 patients with acute ischaemic stroke. Lancet. 1997;349:1641-9.

24. Weaver CS, Leonardi-Bee J, Bath-Hextall FJ, Bath PMW. Sample size calculations in acute stroke trials: a systematic review of their reporting, characteristics, and relationship with outcome. Stroke. 2004;35:1216-24.

25. Dennis M, Wellwood I, Warlow C. Are simple questions a valid measure of outcome after stroke? Cerebrovasc Dis 1997; 7:22-27.

26. Bath PM, Lees KR, Schellinger PD, Altman H, Bland M, Hogg C, Howard G, Saver JL; European Stroke Organisation Outcomes Working Group. Statistical analysis of the primary outcome in acute stroke trials. Stroke. 2012; 43(4):1171-8.

27. Lees KR, Bath PM, Schellinger PD, Kerr DM, Fulton R, Hacke W, Matchar D, Sehra R, Toni D; European Stroke Organization Outcomes Working Group. Contemporary outcome measures in acute stroke research: choice of primary outcome measure. Stroke. 2012; 43(4):1163-70.

28. Rankin J. Cerebral vascular accidents in patients over the age of 60. Scott Med J 1957; 2:200-15.

29. Bonita R, Beaglehole R. Modification of Rankin Scale: Recovery of motor function after stroke. Stroke 1988; 19:1497-1500.

30. Van Swieten JC, Koudstaal PJ, Visser MC, Schouten HJ, van Gijn J. Interobserver agreement for the assessment of handicap in stroke patients. Stroke 1988;19:604-7.

31. Teng EL, Chui HC. The Modified Mini-Mental State (3MS) examination. J Clin Psychiatry. 1987; 48:314-318.

32. Malloy PF, Cummings JL, Coffey CE, Duffy J, Fink M, Lauterbach EC, Lovell M, Royall D, Salloway S. Cognitive screening instruments in neuropsychiatry: a report of the Committee on Research of the American Neuropsychiatric Association. J Neuropsychiatry Clin Neurosci. 1997; 9:189-97.

33. Tombaugh TN, McIntyre NJ. The mini-mental state examination: a comprehensive review. J Am Geriatr Soc. 1992; 40:922-35.

34. Nasreddine ZS, Phillips NA, Bédirian V, Charbonneau S, Whitehead V, Collin I, Cummings JL, Chertkow H. The Montreal Cognitive Assessment, MoCA: a brief screening tool for mild cognitive impairment. J Am Geriatr Soc. 2005; 53:695-9.

35. Pendlebury ST. Cuthbertson FC. Welch SJ. Mehta Z. Rothwell PM. Underestimation of cognitive impairment by mini-mental state examination versus the montreal cognitive assessment in patients with transient ischemic attack and stroke: a population-based study. Stroke. 2010; 41:1290-3.

36. Wen HB, Zhang ZX, Niu FS, Li L. The application of Montreal cognitive assessment in urban Chinese residents of Beijing. Chin J Intern Med. 2008; 47:36-9.

37. Zhang ZX, Plassman BL, Xu Q, Zahner GEP, Wu B, Gai MY, Wen HB, Chen X, Gao S, Hu D, Xiao XH, Shen Y, Liu AM, Xu T. Lifespan influences on mid- to late-life cognitive function in a Chinese birth cohort. Neurology 2009; 73;186-94.

38. Hollander M, Wolfe DA. Nonparametric statistical methods (2nd Edition). New York: John Wiley & Sons. 1999.

39. Klein JP, Moeschberger ML. Survival analysis: techniques for censored and truncated regression. New York, NY: Springer-Verlag; 1997.

APPENDIX A

China Antihypertensive Trial in Acute Ischemic Stroke (CATIS):

Step-wide Treatment Plan

A. Blood Pressure Goals and Timeline:

Blood pressure (BP) lowering will start immediately after randomization. The targeted treatment goals are:

Step 1 (within 24 hours after randomization)

To lower systolic BP by 10 to 25% (but systolic BP not lower than 120 mm Hg and diastolic BP not lower than 80 mm Hg) within the first 24 hours after randomization based on the participant's admission BP levels.

Step 2 (within 7 days after randomization)

To achieve systolic BP below 140 mm Hg and diastolic BP below 90 mm Hg and maintain this BP level afterwards

B. Treatment Algorithm

All participants in the intervention group will receive antihypertensive medications in a staged sequence. Based on patients' baseline BP level, the first-line medication (intravenous angiotensin-converting enzyme inhibitor) can be used alone, or in combination with second-line medication (calcium channel blocker), and third-line medication (diuretics) to achieve the target systolic BP lowering by 10% to 25% within the first 24 hours after randomization according to the following treatment algorithm (figure). The dosage and number of medications will be titrated to achieve systolic BP below 140 mm Hg and diastolic BP below 90 mm Hg within 7 days and maintain this BP level afterwards.

Treatment algorithm for blood pressure reduction group

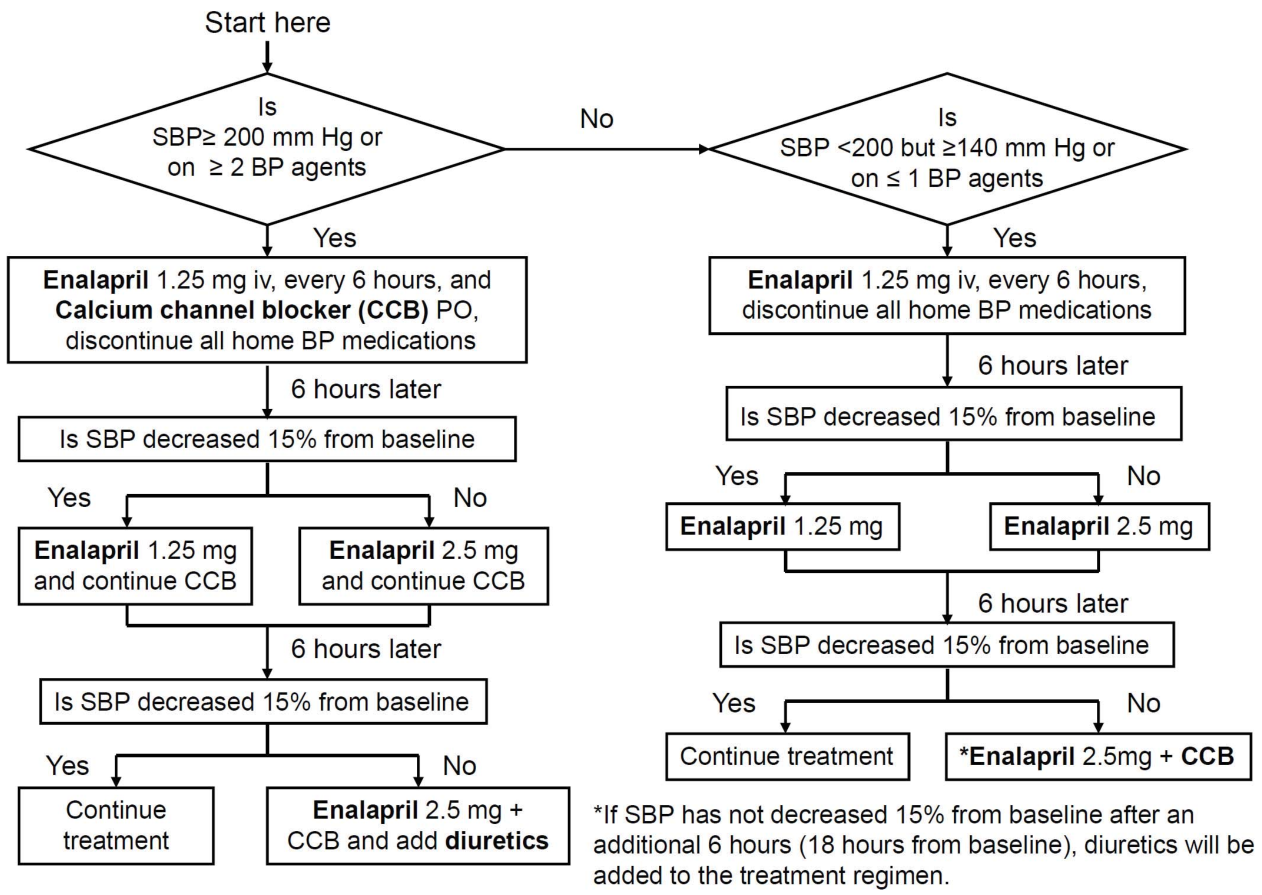

At any step above, if the BP target is achieved, medication will be continued at the same dosage and titrated to maintain the target BP level. If the BP target is not achieved by the above treatment algorithm, medications will be titrated up to the maximum dosage in the order of ACEI, CCB and diuretics before adding another BP agent. Add a 4th agent if needed.

Don't use ACE I, CCB or diuretics if contraindicated.

APPENDIX B

China Antihypertensive Trial in Acute Ischemic Stroke (CATIS):

Data Safety and Monitoring Board (DSMB)

An independent DSMB will oversee the protection of human subjects from research. The DSMB has reviewed and approved this study protocol.

Chair: Paul K. Whelton, M.B., M.D., M.Sc.
Professor of Epidemiology
School of Public Health and Tropical Medicine
Tulane University
New Orleans, LA 70112, USA

Members:
Zhenxin Zhang, MD
Professor and chair
Department of Neurology and Center for Memory Impairment and Movement Disorder
Peking Union Hospital
Peking Union Medical College and Chinese Academy of Medical Sciences
Beijing, China

Xingquan Zhao, MD, PhD
Professor and Vice Chair
Neurology Department
Director, Chinese Stroke Training Center
Tiantan Hospital
Capital Medical University
Beijing, China

Jose Biller, M.D., FACP, FAAN, FAHA
Professor and Chairman

Department of Neurology
Loyola University Chicago
Stritch School of Medicine
2160 South First Avenue
Maywood, IL 60153, USA

C. Lillian Yau
Professor of Biostatistics
East China Normal University
500 Dongchuan Road
Shanghai 200241, China

Amendment #1

October 2009

The CATIS Steering Committee has approved the following change to step 1 (within 24 hours after randomization) of the treatment plan for safety reasons. This change will ensure that patients with a systolic blood pressure of 140 mm Hg will not reduce their systolic blood pressure by more than 10%.

Original text: "To lower systolic blood pressure by 10 to 25% (but systolic blood pressure not lower than 120 mm Hg and diastolic blood pressure not lower than 80 mm Hg) within the first 24 hours after randomization based on the participant's admission blood pressure levels."

Revised text: "To lower systolic blood pressure by 10 to 25% (but systolic blood pressure not lower than 126 mm Hg and diastolic blood pressure not lower than 80 mm Hg) within the first 24 hours after randomization based on the participant's admission blood pressure levels."

Amendment #2

November 2009

The CATIS Steering Committee has approved an ancillary study to study the effect of blood pressure reduction on cognitive impairment in acute ischemic stroke patients. The cognitive impairment will be measured at 3, 12, and 24 months in a subgroup of patients. The modified Mini-Mental State Examination (MMSE) and the Montreal Cognitive Assessment (MoCA) will be used. The MMSE contains 20 items that measure the degree of cognitive impairment in the areas of orientation, concentration, language, praxis, and immediate and delayed memory. The MMSE showed high levels of sensitivity for moderate-to-severe cognitive impairment but lower levels for mild degrees of impairment, whereas MoCA, a brief 30-point global cognitive screen tool, is more sensitive to mild cognitive impairment. Both MMSE and MoCA have been translated into Chinese and validated in the Chinese population.

Amendment #3

August 2011

The CATIS Steering Committee has decided to extend the recruitment period from 24 months to 42 months and to increase the number of clinic sites for recruitment. These changes will be reflected in the annual IRB renewal application.
