# Supplementary material for: Immediate Antihypertensive Treatment for Patients With Acute Ischemic Stroke With or Without History of Hypertension: A Secondary Analysis of the CATIS Randomized Clinical Trial
Source: JAMA Netw Open. 2019 Jul 31;2(7):e198103. doi: 10.1001/jamanetworkopen.2019.8103 (PMC6669782; doi:10.1001/jamanetworkopen.2019.8103)
Supplement: Supplement 2. — eTable 1. Blood Pressure Reduction After Randomization at 14 Days or Hospital Discharge According to History of Hypertension eTable 2. Adjusted Odds Ratios (95% Confidence Intervals) According to History of Hypertension Before Ischemic Stroke Onset eFigure. Treatment Algorithm for Blood Pressure Reduction Group [file jamanetwopen-2-e198103-s002.pdf]

Supplementary Online Content

Zhang R, Zhong C, Zhang Y, et al. Immediate antihypertensive treatment for patients with acute ischemic stroke with or without history of hypertension: a secondary analysis of the CATIS randomized clinical trial. *JAMA Netw Open*. 2019;2(7):e198103. doi:10.1001/jamanetworkopen.2019.8103

eTable 1. Blood Pressure Reduction After Randomization at 14 Days or Hospital Discharge According to History of Hypertension

eTable 2. Adjusted Odds Ratios (95% Confidence Intervals) According to History of Hypertension Before Ischemic Stroke Onset

eFigure. Treatment Algorithm for Blood Pressure Reduction Group

This supplementary material has been provided by the authors to give readers additional information about their work.

© 2019 Zhang R et al. *JAMA Network Open*.

eTable 1. Blood Pressure Reduction After Randomization at 14 Days or Hospital Discharge According to History of Hypertension

	Patients with hypertension				Patients without hypertension			
	Treatment	Control	Difference (95% CI)	*p* value	Treatment	Control	Difference (95% CI)	*p* value
BP at 24 h after randomization, mean (SD), mm Hg								
Systolic	145.1 (15.1)	153.5 (15.8)	-8.4 (-9.5 to -7.3)	<0.001	143.2 (14.5)	150.4 (16.0)	-7.2 (-9.3 to -5.1)	<0.001
Diastolic	86.2 (8.9)	90.0 (9.6)	-3.8 (-4.4 to -3.1)	<0.001	84.7 (8.6)	88.3 (9.7)	-3.7 (-4.9 to -2.4)	<0.001
Absolute BP changed from baseline to 24 h after randomization, mean (SD), mmHg								
Systolic	-21.7 (16.3)	-12.4 (17.4)	-9.3 (-10.5 to -8.2)	<0.001	-22.4 (14.5)	-14.0 (17.1)	-8.4 (-10.5 to -6.2)	<0.001
Diastolic	-10.9 (10.6)	-6.8 (11.1)	-4.1 (-4.9 to -3.4)	<0.001	-11.2 (10.2)	-7.3 (10.6)	-3.9 (-5.3 to -2.5)	<0.001
Proportional BP changes from baseline to 24 h after randomization, mean (SD), %								
Systolic	-12.6 (8.9)	-7.0 (9.8)	-5.6 (-6.3 to -4.9)	<0.001	-13.2 (7.9)	-8.1 (9.8)	-5.1 (-6.3 to -3.9)	<0.001
Diastolic	-10.6 (10.1)	-6.2 (11.1)	-4.4 (-5.1 to -3.6)	<0.001	-11.1 (10.0)	-7.0 (11.0)	-4.0 (-5.5 to -2.6)	<0.001
BP at 7 days after randomization, mean (SD), mm Hg								
Systolic	137.8 (11.9)	147.2 (13.5)	-9.4 (-10.3 to -8.5)	<0.001	135.3 (10.9)	144.0 (13.7)	-8.8 (-10.6 to -7.0)	<0.001

© 2019 Zhang R et al. *JAMA Network Open*.

Diastolic	82.7 (7.2)	86.8 (8.1)	-4.1 (-4.7 to -3.6)	<0.001		81.1 (7.0)	84.7 (7.9)	-3.6 (-4.7 to -2.5)	<0.001
BP at 14 days after randomization, mean (SD), mm Hg									
Systolic	135.4 (10.6)	144.7 (13.8)	-9.3 (-10.6 to -8.0)	<0.001		134.1 (9.8)	139.9 (14.2)	-5.8 (-8.4 to -3.1)	<0.001
Diastolic	81.5 (6.7)	85.8 (8.2)	-4.4 (-5.2 to -3.6)	<0.001		81.3 (10.0)	83.3 (8.4)	-2.0 (-4.0 to 0.1)	0.06

© 2019 Zhang R et al. *JAMA Network Open*.

eTable 2. Adjusted Odds Ratios (95% Confidence Intervals) According to History of Hypertension Before Ischemic Stroke Onset

	Patients with hypertension			Patients without hypertension		*p* value for homogeneity
	Odds ratio (95% CI)	*p* value		Odds ratio (95% CI)	*p* value	
**14 days or hospital discharge**						
Primary outcome of death or major disability	1.03 (0.84-1.26)	0.79		1.19 (0.82-1.74)	0.36	0.40
Ordinal modified Rankin score	0.98 (0.85-1.12)	0.74		1.06 (0.81-1.37)	0.69	0.51
Death	0.75 (0.36-1.55)	0.43		0.50 (0.06-4.38)	0.53	0.84
**3-month post-treatment follow-up visit**						
Composite death or major disability	0.99 (0.80-1.22)	0.91		1.31 (0.87-1.96)	0.20	0.23
Ordinal modified Rankin score	0.99 (0.86-1.14)	0.91		1.12 (0.86-1.45)	0.40	0.47
Death	1.07 (0.66-1.74)	0.78		2.61 (0.98-6.98)	0.06	0.22
Recurrent stroke	0.47 (0.26-0.85)	0.01		3.59 (0.90-14.40)	0.07	0.01
Vascular events	0.68 (0.43-1.08)	0.10		2.14 (0.79-5.83)	0.14	0.05

OR, odds ratio; CI, confidence interval.

© 2019 Zhang R et al. *JAMA Network Open*.

[a] Adjusting for age, sex, baseline SBP, BMI, diabetes, cigarette smoking, stroke subtype, baseline NIHSS score and time from onset to randomization.

© 2019 Zhang R et al. *JAMA Network Open*.

eFigure. Treatment Algorithm for Blood Pressure Reduction Group

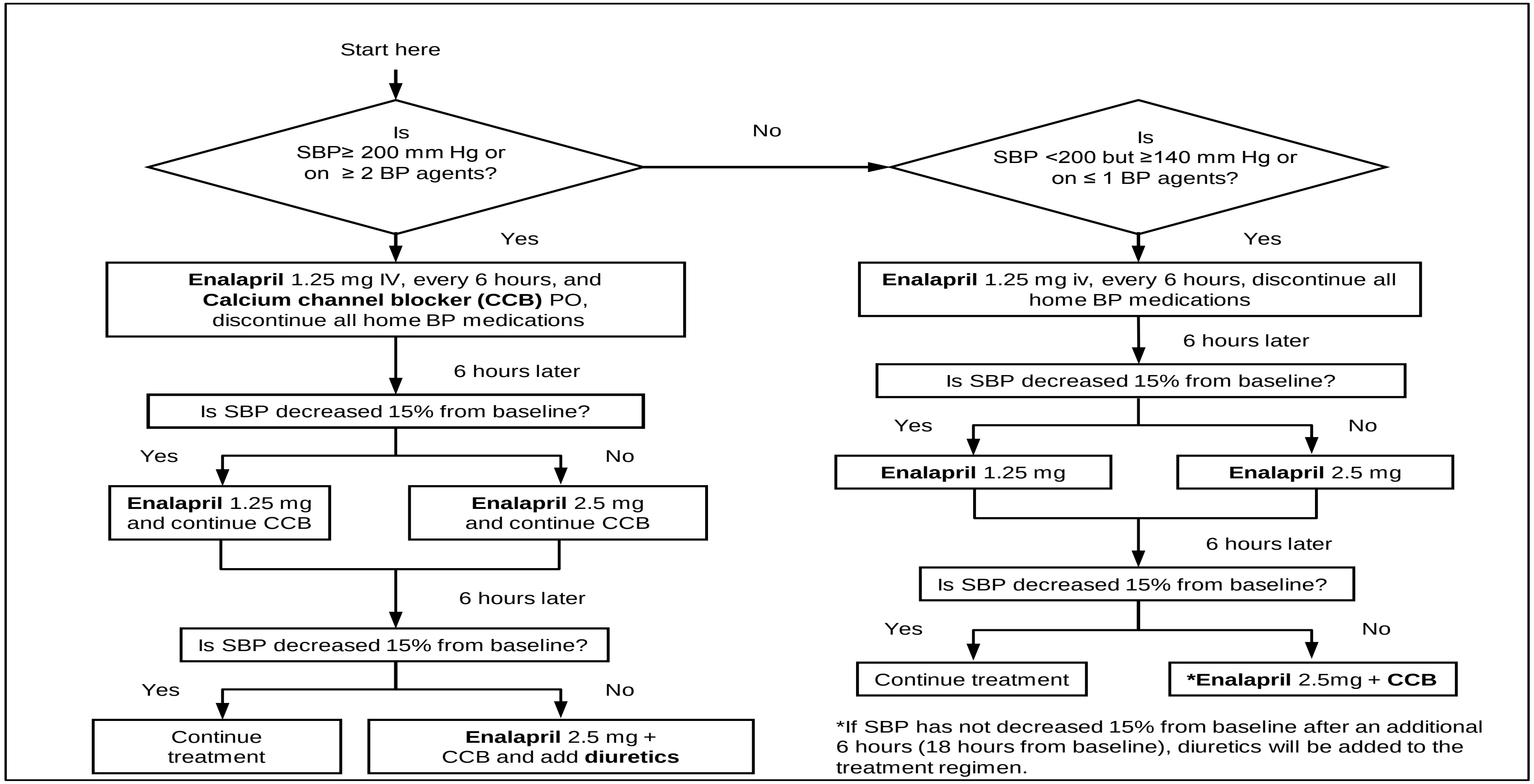

© 2019 Zhang R et al. *JAMA Network Open*.
